# Low blood pressure

**DOI:** 10.1186/1471-2393-12-S1-A9

**Published:** 2012-08-28

**Authors:** Jane Warland

**Affiliations:** 1 University of South Australia, Australia

## 

The link between high maternal blood pressure and poor pregnancy outcome is well established. Similarly the causal relationship between acute maternal hypotension and acute fetal distress is well recognised. The link between poor pregnancy outcome and persistent maternal hypotension is less well known.

McClure Brown [1] was the first to report an increase in perinatal mortality in the presence of persistent maternal hypotension. In a prospective trial involving more than 7,000 primigravid women conducted in the 1960s he noticed a “curious” association between low initial (first visit) systolic and diastolic blood pressure and increased risk of stillbirth. Nearly 20 years later Friedman and Neff [2] also found a similar level of risk in a population based study of more than 38,000 women who presented with persistently low (over 4 visits) diastolic hypotension and poor pregnancy outcome. A number of German studies conducted in the 1980s also variously reported findings suggesting a relationship between maternal hypotension and poor pregnancy outcome such as FGR and stillbirth. For example, a retrospective study comparing hypotensive pregnant women with their normotensive peers found there was an increased risk of preterm birth, IUGR and complications such as meconium stained liquor and post partum hemorrhage in the hypotensive group [3]. There were seven stillbirths in total in their study, four in the hypotensive and three in the normotensive groups, but the study was underpowered to detect any statistically significant difference on this rare outcome. Zhang and Klebanoff [4] further investigated the “paradox” of hypotension. Using the same data bank as Friedman and Neff they found the lower the baseline the higher the risk of poor pregnancy outcome. They suggested that the mechanism for this finding may be poor placental perfusion in the hypotensive group, also implicated in several of the German studies. In another large population based study Steer et al [5] described higher risk of low birth weight and increased risk of perinatal mortality in their hypotensive group. Although Chen [6] pointed out that this finding may be due to failure to take into account gestational length, in a case-control study matched for gestational age [7] still found an association between hypotension and stillbirth.

It is problematic that previously reported studies have defined maternal hypotension differently. However, Warland [7] attempted to address this by including all previously used definitions as well as mean arterial pressure (MAP). Diastolic hypotension and low MAP seemed more “dangerous” than systolic hypotension and this supports findings from earlier German studies which suggest that the casual mechanism for hypotension on poor pregnancy outcome is poor placental perfusion.

In summary, there is a small body of research which has consistently demonstrated the negative effect of persistent maternal hypotension on poor pregnancy outcome including stillbirth. These studies have been conducted using a range of approaches including prospective cohort, retrospective case-control and population based data bank analysis. Many questions remain unanswered, including the definition of hypotension, the level at which hypotension becomes problematic, and how best to manage maternal hypotension in pregnancy.
